# Current status of liver transplantation for non‐B non‐C liver cirrhosis and hepatocellular carcinoma

**DOI:** 10.1002/ags3.12612

**Published:** 2022-08-23

**Authors:** Takahiro Nishio, Takashi Ito, Koichiro Hata, Kojiro Taura, Etsuro Hatano

**Affiliations:** ^1^ Department of Surgery, Graduate School of Medicine Kyoto University Kyoto Japan

**Keywords:** alcoholic liver disease, liver transplantation, nonalcoholic fatty liver disease, nonalcoholic steatohepatitis, non‐B non‐C hepatocellular carcinoma

## Abstract

Recently, non‐B non‐C chronic liver diseases, including alcoholic liver disease (ALD) and nonalcoholic fatty liver disease (NAFLD)/nonalcoholic steatohepatitis (NASH), have markedly increased worldwide. Liver transplantation (LT) is an effective curative therapy for hepatocellular carcinoma (HCC) as well as decompensated liver cirrhosis. In Japan, where the source of liver grafts is strongly dependent on living donors, efforts have been made to unify the indications for eligibility of HCC patients for LT, leading to the development of 5‐5‐500 criteria. Along with the expansion of eligibility for LT, the current changing trends in underlying liver diseases of LT recipients, which are related to the rising tide of non‐B non‐C cirrhosis and HCC, are highlighting the importance of peri‐transplant management of patients with various comorbidities. The post‐LT prognosis of patients with ALD is significantly affected by de novo malignancies and metabolic syndrome‐related complications as well as posttransplant alcohol relapse. NAFLD/NASH patients often suffer from obesity, type 2 diabetes mellitus, and other metabolic syndrome‐related disorders, and nonneoplastic factors such as cardiovascular events and recurrence of NAFLD/NASH have a significant impact on post‐LT outcomes. Patient management in the peri‐transplant period as well as risk assessment for LT are key to improving post‐LT outcomes in the era of a growing number of cases of LT for non‐B non‐C liver diseases.

## Graphical abstract

Recently, the cases of LT for non‐B non‐C cirrhosis and HCC have been increasing in Japan as well as worldwide. Perioperative management of non‐B non‐C liver disease is problematic due to various risk factors, including alcohol consumption and metabolic syndrome‐related risks, such as obesity and diabetes mellitus. This article aims to review the current status and issues of LT focusing on non‐B non‐C liver diseases, including HCC." cd_value_code="text

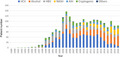

## 1 INTRODUCTION

Hepatocellular carcinoma (HCC) often develops in the setting of chronic liver injury. Liver transplantation (LT) for HCC is an effective curative therapy that can simultaneously replace not only the tumors but also the damaged liver parenchyma from which the tumors originate. In recent years, there has been a global shift in the causes of chronic liver injury and the resulting HCC, leading to changing trends in the management of LT.^1^ The number of patients with chronic infection of hepatitis B virus (HBV) or hepatitis C virus (HCV), which used to be the main cause of decompensated cirrhosis and HCC, has been decreasing due to advances in antiviral therapy using nucleic acid analogs for HBV and direct acting antivirals (DAAs) for HCV. Alternatively, non‐B non‐C liver cirrhosis and HCC, which arise from hepatitis virus‐unrelated chronic liver diseases, including alcoholic liver disease (ALD) and nonalcoholic fatty liver disease (NAFLD)/nonalcoholic steatohepatitis (NASH), are markedly increasing.^2^, ^3^ NAFLD/NASH patients have a high prevalence of obesity, type 2 diabetes mellitus, and other metabolic syndrome‐related disorders,^4^, ^5^, ^6^ and nonneoplastic factors such as cardiovascular events and NAFLD/NASH recurrence have a significant impact on post‐LT outcomes. Regarding patients with ALD, not only posttransplant alcohol relapse but also de novo malignancies and metabolic syndrome‐related comorbidities are important prognostic factors that are potentially problematic in peri‐transplant management.^7^, ^8^

The causes of liver injury and systemic comorbidities that underlie non‐B non‐C liver diseases are diverse, frequently overlapping and further complicating the prognostic impact after LT. In addition, the concept of metabolic dysfunction‐associated fatty liver disease (MAFLD) has been proposed as a fatty liver disease associated with metabolic dysfunction due to obesity, diabetes, and metabolic syndrome‐related abnormalities, which is independent from the conventional definition of alcoholic intake.^9^ As patients with non‐B non‐C liver diseases increase along with the rising prevalence of metabolic disorders, MAFLD will gain more attention as an underlying etiology of LT recipients, which may significantly impact their post‐LT prognosis.

Thus, patient management and education in the peri‐transplant period as well as the indication and risk assessment for LT are key to improving the post‐LT outcomes in the era of a growing number of cases of LT for non‐B non‐C etiologies. This article aims to review the current status and issues of LT focusing on non‐B non‐C liver diseases, including HCC.

## 2 INDICATIONS FOR LIVER TRANSPLANTATION FOR HCC IN JAPAN

LT provides a good treatment option for selected patients with HCC, achieving long‐term survival without cancer recurrence due to efforts to establish appropriate indications. In 1996, Mazzaferro et al proposed the Milan criteria (single lesion ≤5 cm; ≤3 lesions all ≤3 cm; no extrahepatic metastasis or macrovascular invasion), which has been widely accepted as a global gold standard.^10^ Subsequently, because there are a number of cases that do not meet the Milan criteria but have good results as well as cases that meet the criteria but show a high risk of recurrence (10%‐16%),^11^ attempts have been made to develop novel criteria that could more accurately assess the risk of HCC recurrence in patients and to expand eligibility for LT in patients with HCC beyond Milan criteria (Table 1).

**TABLE 1:** Liver transplantation selection models for patients with HCC

	Year	Tumor burden	Biomarker	Additional criteria	Post‐LT survival	C‐index
DDLT						
Milan criteria^10^	1996	Single tumor ≤5 cm or 3 tumors all ≤3 cm	N.A.	N.A.	4‐year OS 85% (within criteria) vs 50% (beyond criteria)	N.A.
UCSF criteria^12^, ^13^	2001 2007	Single tumor ≤6.5 cm or 3 tumors all ≤4.5 cm with TTD ≤8 cm	N.A.	N.A.	5‐year OS 75.2% (within criteria) in [12] 5 year RFS 80.9% (within criteria) in [13]	N.A.
Up‐to‐7^15^	2009	The sum of the maximum tumor diameter and number <7	N.A.	N.A.	5‐year OS 71.2% (beyond Milan; within Up‐to‐7) vs 48.1% (beyond Milan and Up‐to‐7)	N.A.
French AFP model^16^	2012	Size (≤3, 3‐6, >6 cm) and number (1‐3, ≥4)	AFP (≤100, 100‐1000, >1000 ng/mL)	N.A.	5‐year OS 69.9%, 5‐year RR 13.4% (score ≤2) vs 5‐year OS 40.8%, 5‐year RR 45.3% (score >2)	0.70
Total tumor volume (TTV)/AFP^17^	2015	Total tumor volume ≤115 cm^3^	AFP ≤400 ng/mL	N.A.	4‐year OS 74.6% (beyond Milan; within TTV/AFP) vs 78.7% (within Milan)	N.A.
HALT‐HCC^a^, ^18^	2017	TBS^b^: hypotenuse between tumor number and largest tumor size	lnAFP	MELD‐Na	5‐year OS 78.7% (quartile 1), 74.5% (quartile 2), 71.8% (quartile 3), 61.5% (quartile 4)	0.61
Metroticket 2.0^c^, ^19^	2018	Tumor number + size (in cm) of the largest tumor	AFP (<200, 200‐400, 400‐1000, >1000 ng/mL)	N.A.	5‐year OS 79.7%, 5 years RFS 89.6% (within criteria) vs 5‐year OS 51.2%, 5 years RFS 46.8% (beyond criteria)	0.72
LiTES‐HCC^34^	2021	TTD at listing and at transplant	Initial AFP, Pretransplant AFP	age, bilirubin, INR, CKD, eGFR, etiology (HCV/HBV/NASH/Alcohol/Other), ventilation, pretransplant location (home/hospital/ICU)		0.62
LDLT (Japan)						
Tokyo criteria^28^	2007	≤5 tumors, largest tumor ≤5 cm	N.A.	N.A.	3‐year RFS 94% (within criteria) vs 50% (beyond criteria)	N.A.
Kyoto criteria^30^	2007	≤10 tumors, largest tumor ≤5 cm	DCP ≤400 mAU/mL	N.A.	5‐year OS 87%, 5‐year RR 5% (within criteria) vs 5‐year OS 37%, 5‐year RR 61% (beyond criteria)	N.A.
A‐P criteria^31^	2007	Milan criteria	AFP ≤200 ng/mL, DCP ≤100 mAU/mL	N.A.	5‐year RFS 99.5% (within Milan; within A‐P), 84.3% (beyond Milan; within A‐P), 85.0% (within Milan; beyond A‐P), 45.0% (beyond Milan; beyond A‐P)	N.A.
Kyushu criteria^32^	2011	No limit tumor number, largest tumor <5 cm	DCP <300 mAU/mL	N.A.	5‐year RFS 80.0% (within criteria) vs 0% (beyond criteria)	N.A.
5‐5‐500 criteria^33^	2019	≤5 tumors, largest tumor ≤5 cm	AFP ≤500 ng/mL	N.A.	5‐year OS 75.8%, 5 years RFS 73.2% (within criteria) vs 5‐year OS 52.1%, 5‐year RFS 43.4% (beyond criteria)	N.A.
Japan criteria^33^	2019	Milan criteria or 5‐500 criteria	AFP ≤500 ng/mL	N.A.	5‐year OS 74.8%, 5‐year RFS 71.8% (within criteria) vs 5‐year OS 48.6%, 5‐year RFS 40.0% (beyond criteria)	N.A.

Abbreviations: AFP, alpha fetoprotein; AUC, area under the curve; CKD, chronic kidney disease; DCP, des‐γ‐carboxy prothrombin; DDLT, deceased donor liver transplantation; eGFR, estimated glomerular filtration rate; HBV, hepatitis B virus; HCC, hepatocellular carcinoma; HCV, hepatitis C virus; ICU, intensive care unit; INR, international normalized ratio of prothrombin time; LDLT, living donor liver transplantation; LT, liver transplantation; MELD, model for end‐stage liver disease; NASH, nonalcoholic steatohepatitis; OS, overall survival; RFS, recurrence‐free survival; RR, recurrence rate; TBS, tumor burden score; TTD, total tumor diameter; TTV, total tumor volume.

^a^ HALT‐HCC = (1.27 × TBS) + (1.85 × lnAFP) + (0.26 × MELD‐Na)

^b^ TBS^2^ = (tumor number)^2^ + (largest tumor diameter)^2^.

^c^ AFP‐adjusted‐to‐HCC‐size criteria of transplantability: HCC within the Up‐to‐7 criteria, if AFP <200 ng/mL; HCC within the Up‐to‐5 criteria, if AFP 200‐400 ng/mL; HCC within the Up‐to‐4 criteria, if AFP 400‐1000 ng/mL (considering Up‐to‐7, ‐5, or ‐4 as the maximum allowed sum of size (in cm) and number of tumors derived in any given HCC before transplantation regardless of whether preceded by neoadjuvant therapies).

Similar to the Milan criteria, tumor burdens have been principally used for indications with modest modification, including the University of California San Francisco (UCSF) criteria,^12^, ^13^ Total Tumor Volume (TTV),^14^ and Up‐to‐7.^15^ For further expansion of eligibility, the cancer biological marker alpha‐fetoprotein (AFP) was combined with tumor burdens, leading to proposals such as the AFP model,^16^ TTV/AFP,^17^ HALT‐HCC,^18^ and Metroticket 2.0.^19^ Furthermore, incorporating FDG‐PET^20^ and the neutrophil/lymphocyte ratio (NLR)^21^ was suggested to assess the risk of post‐LT tumor recurrence. Predictive models to assess the malignant potential and the risk of post‐LT recurrence based on histopathological findings (degree of differentiation, microvascular invasion) of the excised liver (post‐MORAL,^22^ RETREAT score^23^) were reported, thus providing useful information for therapeutic strategies after LT. In addition, the effectiveness of downstaging by locoregional therapy prior to LT for patients exceeding the selection criteria and incorporating the pre‐LT therapeutic response has been proposed (UNOS‐DS).^24^, ^25^

In Japan, unlike in Europe and the United States, living donor (LD) is the mainstay of donor sources for LT due to the critical shortage of deceased donor livers, and thus the eligibility of patients with HCC for LT are limited to those with decompensated cirrhosis.^26^ LDLT potentially requires ethical, medical, and surgical considerations compared to cadaveric LT, and clinical recommendations for specific approaches to LDLT are proposed by the guidelines of the International Liver Transplantation Society (ILTS).^27^ In efforts to expand the indications for HCC chiefly in the settings of LDLT, several high‐volume institutions in Japan have validated indices beyond the Milan criteria, which incorporated modified tumor burdens (Tokyo Criteria^28^, ^29^) and biological markers, including AFP, des‐gamma‐carboxy prothrombin (DCP) (Kyoto criteria,^30^ A–P criteria,^31^ and Kyushu criteria^32^). Against this background, an attempt was made to unify the criteria for eligibility of HCC patients for LT based on an analysis of the results of nationwide data collection in Japan, leading to the development of the 5‐5‐500 criteria (tumor diameter 5 cm or less, tumor number 5 or less, and AFP 500 ng/mL or less).^33^ The Japan criteria, within the 5‐5‐500 rule OR the Milan criteria, have gained acceptance as a standard indication for LT in patients with HCC.

Thus, efforts to expand the indications of LT for patients with HCC and to improve the outcomes by reducing the risk of tumor recurrence have established liver transplantation as one of the curative therapeutic options for HCC. Alternatively, most of the abovementioned criteria incorporated only HCC‐specific factors and did not sufficiently take into account outcomes other than cancer recurrence. In particular, the prognosis of non‐B non‐C HCC with underlying diseases such as ALD and NAFLD/NASH might be affected by cardiovascular diseases, de novo malignancies, and other etiology‐related sociomedical backgrounds of patients. Recent reports have proposed a prognostic model after liver transplantation for HCC that takes into consideration not only HCC‐related variables but also non‐HCC‐specific factors such as age, renal function, and etiology (LiTES‐HCC score^34^). In Europe and the United States, more than 20% of LT recipients are over the age of 65.^8^, ^35^ In Japan, being faced with a remarkably aging society, the mean age of HCC patients is rising along with the increase in non‐B non‐C HCC,^2^, ^36^ and it will be an issue whether the conventional recipients' age limit of 65 years should be appropriate, whereas the increasing number of eligible patients are elderly. The expansion of indications of LT for patients with HCC may highlight the need for assessing various risk factors associated with the rising trend of non‐B non‐C liver diseases.

## 3 LIVER TRANSPLANTATION FOR DECOMPENSATED CIRRHOSIS AND HCC IN JAPAN—FROM THE REGISTRY BY THE JAPANESE LIVER TRANSPLANTATION SOCIETY (REF. 37)

**FIGURE 1:**
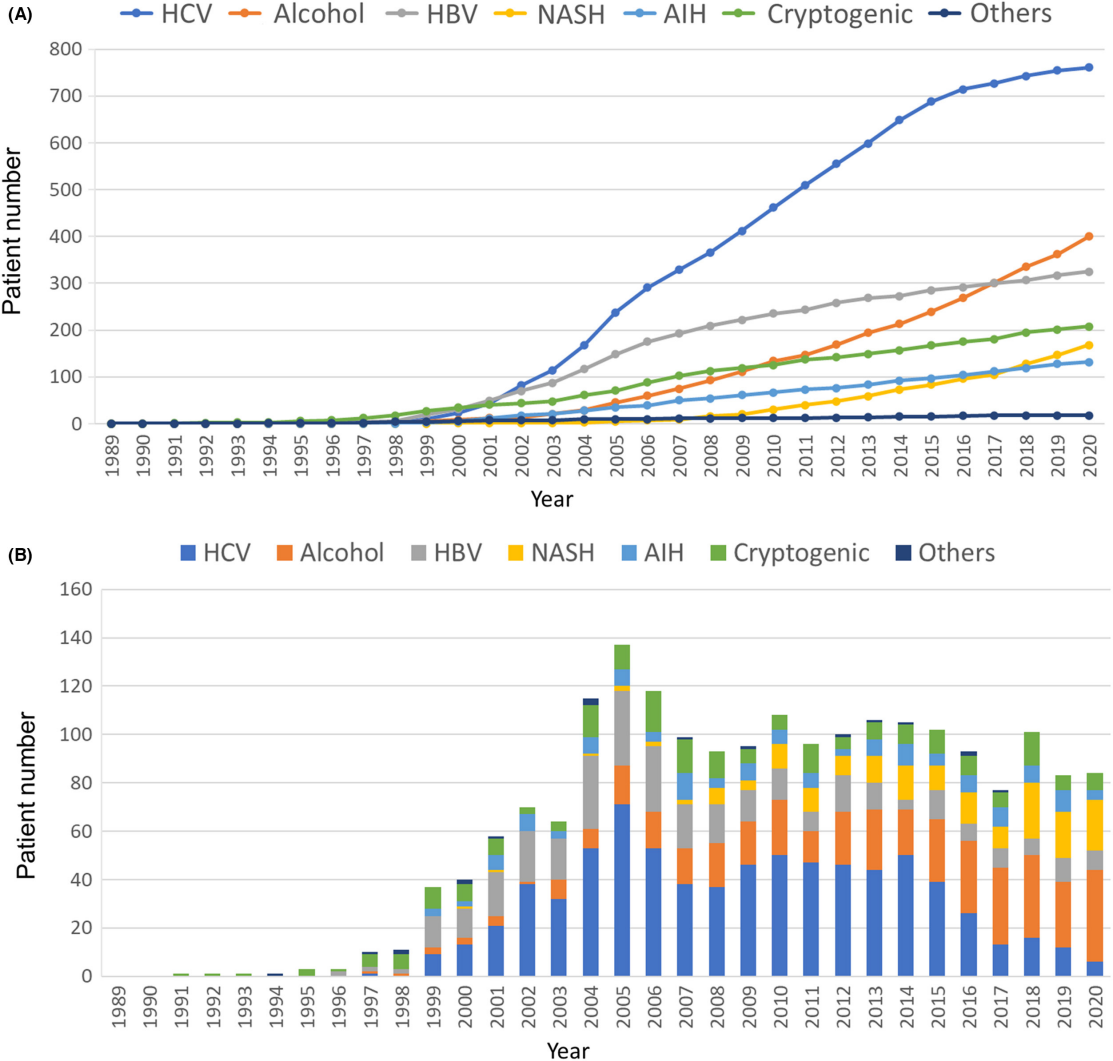
Living donor liver transplantation for hepatocellular diseases without HCC (1989‐2020). (A) Cumulative number of cases and (B) annual trends based on the etiologies of hepatocellular diseases without HCC (reproduced with permission; ref. 36: The Japanese liver transplantation society. Liver transplantation in Japan―registry by the Japanese liver transplantation society. Ishoku 2021;56(3):217–233). AIH, autoimmune hepatitis; HBV, hepatitis B virus; HCC, hepatocellular carcinoma; HCV, hepatitis C virus; NASH, nonalcoholic steatohepatitis.

**FIGURE 2:**
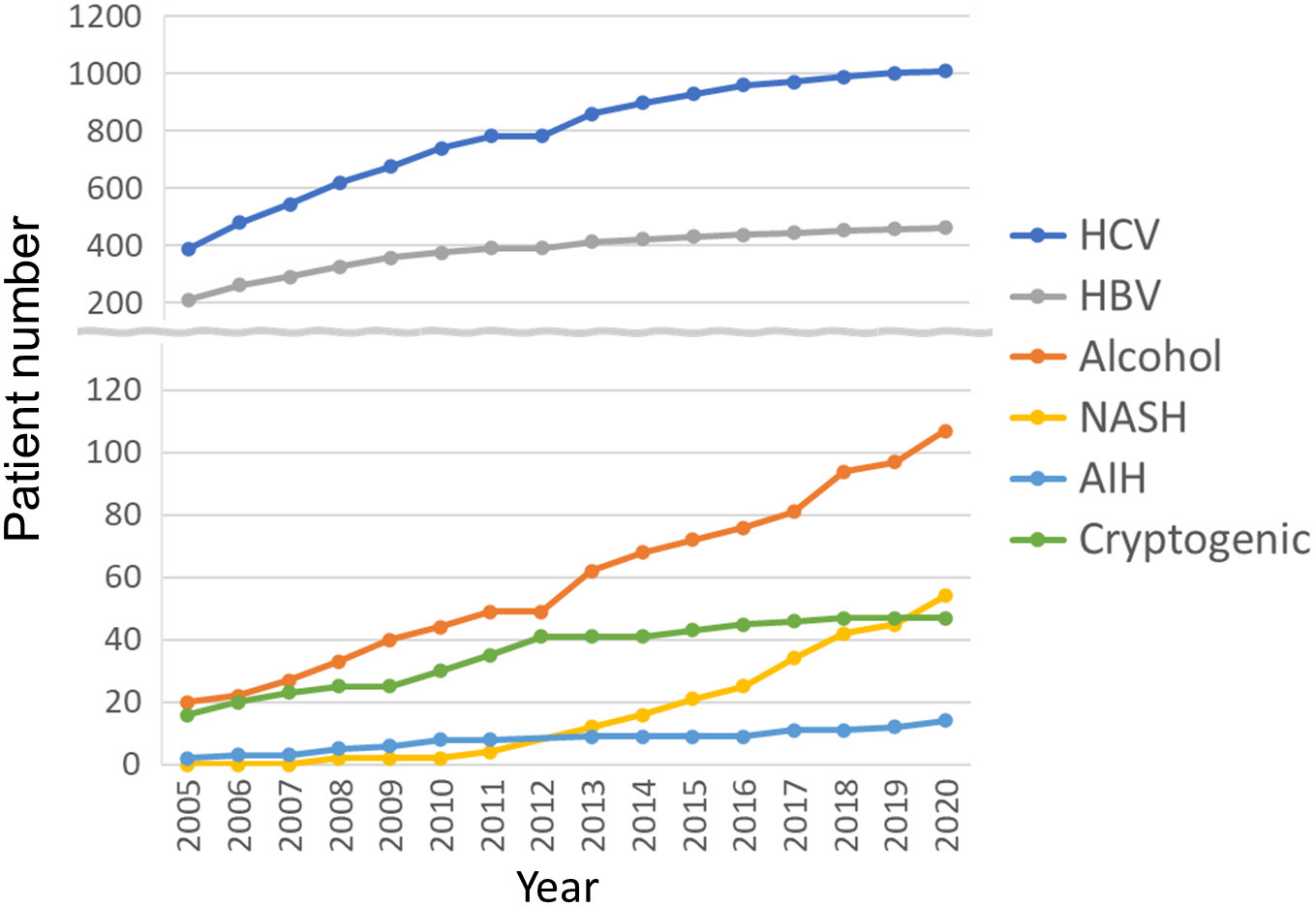
Living donor liver transplantation for hepatocellular diseases with HCC. Annual trends in the cumulative number of cases based on the etiologies from 2005‐2020 are shown (reproduced with permission; ref. 36: The Japanese liver transplantation society. Liver transplantation in Japan―registry by the Japanese liver transplantation society. Ishoku 2021;56(3):217–233). AIH, autoimmune hepatitis; HBV, hepatitis B virus; HCC, hepatocellular carcinoma; HCV, hepatitis C virus; NASH, nonalcoholic steatohepatitis.

According to the 2020 annual report of Liver Transplantation in Japan, registry by the Japanese Liver Transplantation Society,^37^ 2012 cumulative cases of LDLT for hepatocellular diseases without HCC (non‐HCC) were composed of 761 (37.8%) HCV patients, 400 (19.9%) ALD, 325 (16.2%) HBV, 168 (8.3%) NASH, 132 (6.6%) autoimmune hepatitis (AIH), and 208 (10.3%) cryptogenic cirrhosis. Focusing on annual trends, HBV‐ and HCV‐related cirrhosis showed a declining trend, while cases with ALD and NASH markedly increased (Figure 1). The cumulative number of HCC cases was 1747, including 1007 (57.6%) HCV patients, 461 (26.4%) HBV, 107 (6.1%) ALD, 54 (3.1%) NASH, 30 (1.7%) primary biliary cholangitis (PBC), 14 (0.8%) AIH, and 49 (2.8%) cryptogenic. Although viral‐related HCC accounted for the majority of background liver diseases in HCC, the recent increase in ALD‐ and NASH‐related HCC was conspicuous (Figure 2). The 1‐, 3‐, 5‐, and 10‐year overall survival rates after LDLT for non‐HCC patients were 82.9%, 79.1%, 76.2%, and 67.7%, respectively, and those for HCC patients were 84.9%, 76.1%, 70.9%, and 63.1%, respectively. Regarding the etiologies of non‐HCC cirrhosis, the 1‐, 3‐, 5‐, and 10‐year posttransplant survival rates were 79.7%, 74.7%, 71.5%, and 63.2% for HCV; 85.9%, 82.7%, 77.9%, and 62.4% for ALD; 86.1%, 82.5%, 81.5%, and 75.6% for HBV; 87.2%, 84.8%, 81.6%, and 69.0% for NASH; 81.8%, 80.9%, 80.9%, and 78.2% for AIH; and 82.2%, 78.6%, 75.1%, and 68.3% for cryptogenic cirrhosis, respectively.

Similar to the global trends, the cases of LT for non‐B non‐C liver diseases have been increasing in Japan, highlighting the importance of peri‐transplant risk assessment and management for such patients with various comorbidities. Here, we describe the clinical issues regarding LT for non‐B non‐C cirrhosis and HCC, specifically focusing on ALD and NAFLD/NASH, the two major causes of non‐B non‐C liver diseases that show marked increase worldwide and raise growing concerns about their clinical management.

## 4 LIVER TRANSPLANTATION FOR ALD‐RELATED CIRRHOSIS AND HCC

ALD‐related cirrhosis is the second most common hepatocellular disease among LT recipients following HCV in Japan, showing a marked increase in recent years.^37^ In determining the indication of LT for patients with ALD, it is essential to predict the risk of posttransplant alcohol relapse and to provide patient education and support to prevent relapse in the peri‐transplant period. Globally, the 6‐month rule, which requires at least 6 months of abstinence prior to LT, has been accepted as an indication; however, an accurate predictor of the risk of post‐LT alcohol relapse is still controversial.^38^ An analysis of LT for patients with ALD in Japan showed that neither the 6‐month period of abstinence nor the high‐risk alcoholism relapse scale contributed to the risk of harmful relapse, concluding the importance of sociomedical support for patients with alcohol abuse in the perioperative period.^39^ Analysis of the duration of abstinence showed that abstinence for less than 18 months prior to LT was associated with harmful relapse.^40^ In Japan, where the resource of deceased‐donor livers is limited, 18 months of abstinence prior to LT has been accepted as an indication for deceased‐donor LT, while the 6‐month rule is commonly used as a standard indication for LDLT.

Excessive alcohol consumption is associated with 30%‐50% of HCCs in Western countries, the cases of which are increasing as the proportion of viral‐related HCC is declining.^38^ In Japan, ALD‐related HCC was reported to account for 30%‐50% of non‐B non‐C HCCs, which constituted 25%‐30% of HCC.^2^ The problem with ALD is that the risk of carcinogenesis persists long after abstinence and that even moderate alcohol consumption (daily ethanol 20‐70 g/d for females; 30‐70 g for males) is a risk factor for HCC development.^2^, ^6^ Furthermore, patients with ALD are likely to have metabolic syndrome‐related comorbidities such as obesity and diabetes mellitus,^41^, ^42^ which might also contribute to hepato‐carcinogenesis.^43^

The prognosis of LT for ALD in Europe and the United States is not significantly different from that for other indications.^44^ The outcomes of LDLT in Japan showed a slightly better survival rate for ALD than for HCV according to the abovementioned national registry, with no significant difference.^37^ The prognosis of patients with ALD was more likely to be impacted by de novo malignancy and cardiovascular disease than other chronic liver diseases.^45^, ^46^ ALD patients after LT are known to be exposed to a 1.5‐ to 2‐fold risk of developing aerodigestive cancers.^47^ In addition to alcohol consumption itself, various factors, such as advanced age, high smoking rates, obesity and related metabolic abnormalities, and the use of immunosuppressants might contribute to the high risk for developing malignancies in patients with ALD.^48^

Post‐LT alcohol relapse also has a significant impact on outcomes, such as early recurrence of alcoholic steatohepatitis or cirrhosis, acute rejection, and poor immunosuppressant adherence that leads to graft failure.^49^, ^50^, ^51^ Severe psychiatric comorbidities, such as depression, posttraumatic stress disorder, and chronic pain, can also be related to post‐LT mortality.^44^ In a long‐term follow‐up, approximately half of the patients are estimated to show any type of alcohol relapse, which is associated with not only the duration of drinking prior to LT but also with a family history of alcohol abuse and insufficient social support. Early intervention with appropriate sociomedical support might be effective, including smoking cessation programs and depression treatment as well as abstinence from alcohol.^52^ A multidisciplinary team approach consisting of behavioral therapy and pharmacotherapy is critical to improve the outcome of ALD recipients after LT, enabling the integration of hepatology and addiction.^53^ LT programs generally include completion of intensive behavioral therapy followed by regular attendance in Alcoholics Anonymous meetings prior to listing. Behavioral modalities such as cognitive behavioral therapy (CBT) and motivational enhancement therapy (MET) help utilize in‐person social networking with encouragement to attend Alcoholics Anonymous meetings.^54^ A systematic review showed that a combination of CBT, MET, and comprehensive medical care significantly increased alcohol abstinence.^53^, ^55^

## 5 LIVER TRANSPLANTATION FOR NAFLD/NASH‐RELATED CIRRHOSIS AND HCC

While LT for HCV‐related HCC has been declining in Europe and the United States with the advent of DAAs, NAFLD/NASH‐related HCC is the most rapidly growing, and NAFLD/NASH is expected to replace HCV as the main indication of LT for HCC.^8^ The development of NAFLD/NASH is closely associated with advanced age, obesity, diabetes, dyslipidemia, and cardiovascular disease. A high body mass index of recipients is known to worsen overall and graft survival after LT.^56^ Weight reduction protocols and bariatric surgery are beneficial for recipients with severe obesity in Western countries.^57^ In Japan, the number of LT recipients with NAFLD/NASH‐related HCC is steadily increasing, and the management of metabolic complications in the perioperative period is becoming an important issue to address.

Regarding prognosis, the overall survival of NAFLD/NASH‐related HCC after LT is not significantly different compared to other underlying diseases (1‐, 5‐, and 10‐year survival; NASH‐HCC, 89%, 69%, and 47% vs non‐NASH‐HCC, 87%, 58%, and 63%, respectively).^58^ Additionally, post‐LT tumor recurrence rates are similar between NASH and non‐NASH etiologies.^59^ Whereas deaths from extrahepatic malignancy or recurrent liver disease are less frequent, NAFLD/NASH is associated with a higher rate of cardiovascular mortality than non‐NASH etiologies.^60^ Renal failure due to metabolic syndrome significantly affects overall survival and cardiovascular mortality, and other metabolic disorder‐related risks, such as sarcopenia and sarcopenic obesity, are also known prognostic factors of NAFLD/NASH recipients.^61^

Considering the significantly high risk of cardiovascular events in recipients with NAFLD/NASH, pre‐LT evaluation and management of metabolic risk factors, including central obesity, type 2 diabetes, hypertension and dyslipidemia, are of key importance. Screening of cardiovascular and renal function is also recommended.^62^, ^63^ There is no evidence that specific management is required for LT recipients with NAFLD/NASH, who are mostly treated in the same manner as the general population with optimal control of individual components of metabolic syndrome and cardiovascular risk factors.^64^, ^65^

In patients with NAFLD/NASH who have persistent risks of metabolic syndrome, it is important to adopt strategies of management and treatment that focus on improving and controlling the metabolic complications depending on the individual risk factors while avoiding increasing the metabolic risk factors by appropriate immunosuppressive regimens. High‐dose corticosteroids are associated with the risk for metabolic syndrome, and protocols for early reduction and withdrawal of steroids are preferable.^66^ The risk of developing or worsening diabetes was reportedly higher with tacrolimus than with cyclosporine.^67^, ^68^, ^69^ For the management of diabetes, immunosuppressive drug modulation is recommended, including early tapering of steroids and minimization of CNIs by adding antimetabolites or mammalian target of rapamycin inhibitors (mTORi). Patients with NAFLD‐related cirrhosis may benefit from the use of oral anti‐diabetic drugs, including thiazolidinediones, glucagon‐like peptide‐1 receptor agonists, and possibly dipeptidyl peptidase‐4 inhibitors or sodium‐glucose cotransporter‐2 inhibitors.^62^, ^66^, ^70^ Post‐LT hypertension occurs with CNIs and more commonly with cyclosporine,^71^ and modulation of immunosuppressive therapy with minimization of CNIs by adding antimetabolites or mTORi should be considered in patients with arterial hypertension. In addition to lifestyle modification, such as a low‐sodium diet and cessation of smoking, angiotensin‐converting enzyme inhibitors and angiotensin receptor blockers have multiple potential benefits, including proteinuria reduction, afterload reduction, and possible antifibrotic effects. Beta blockers may benefit those with heart failure or coronary artery disease.^66^, ^72^, ^73^ The mTORi is known to have a side effect of dyslipidemia, particularly hypertriglyceridemia.^71^ Minimization protocols of mTORi are preferable for LT recipients with dyslipidemia. Lifestyle modification is the first‐line treatment. In patients not responding to lifestyle improvements, statins represent the first‐line medication option. Patients with isolated post‐LT hypertriglyceridemia will benefit from fibrate and omega‐3 fish oil, especially individuals at a high risk of cardiovascular diseases.^62^, ^66^, ^71^, ^74^ With the goal of minimizing the use of immunosuppressants, caution should be taken in metabolic‐related complications associated with long‐term use of immunosuppressants and their interaction with medications for metabolic disorders. Early use of a renal‐sparing immunosuppressive regimen that minimizes calcineurin inhibitor (CNI) exposure by inducing mammalian target of rapamycin inhibitors (mTORi) is recommended in cases of concomitant renal dysfunction.^61^, ^62^, ^74^, ^75^

Recurrence of NAFLD/NASH is often problematic after LT, and it is important to control weight gain and metabolic syndrome‐related risk factors in the peri‐transplant period. NAFLD recurrence and de novo NAFLD (1‐, 3‐, and 5‐year recurrence rates of 59%, 57%, and 82% and 67%, 47%, and 78%, respectively) are seen in more than half of patients at 1 year after LT. NASH recurrence also occurs frequently (1‐, 3‐, and 5‐year recurrence rates of 53%, 57%, and 45%, respectively), while the incidence of de novo NASH is reportedly relatively low (1‐, 3‐, and 5‐year recurrence rates of 13%, 16%, and 17%, respectively).^61^, ^76^ The post‐LT body mass index and hyperlipidemia were the most consistent predictors of post‐LT graft steatosis.^76^ In addition, donor graft steatosis and genetic predisposition, such as recipient and/or donor PNPLA3, TM6SF2, GCKR, MBOAT7 or ADIPOQ gene polymorphisms, have been shown to be risk factors for recurrent NASH.^77^, ^78^, ^79^ Post‐LT NASH is a strong predictor of long‐term mortality, whereas simple steatosis is unlikely to have a severe impact on post‐LT outcomes.^80^ Standard biochemical liver tests and imaging modalities, including transient or magnetic resonance elastography, are used for monitoring patients at risk of recurrent or de novo NAFLD/NASH after LT.^62^

**FIGURE 3:**
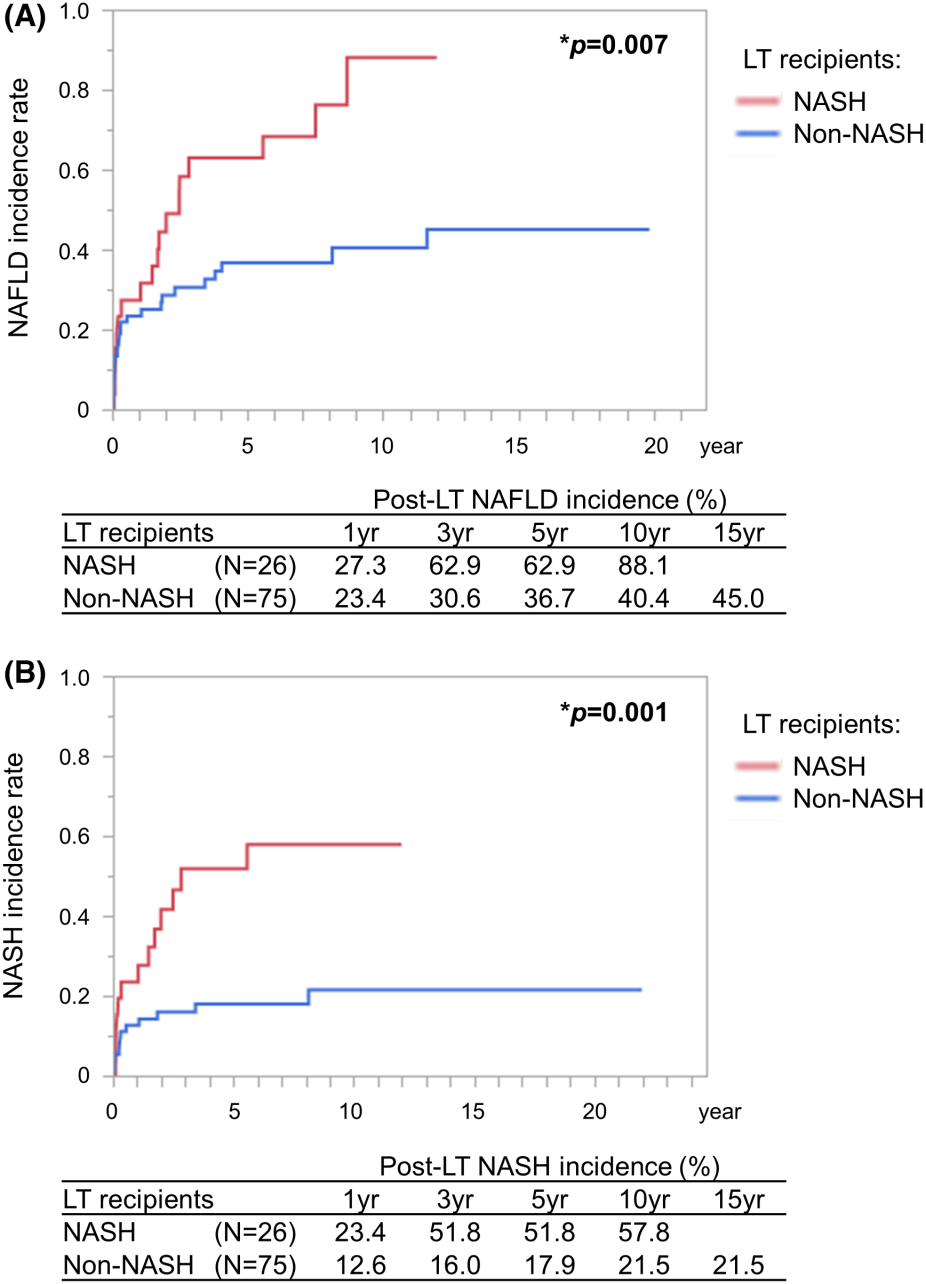
Recurrent or de novo NAFLD/NASH after LT for non‐B non‐C liver disease. One hundred and one recipients who underwent LT for non‐B non‐C cirrhosis and HCC from 1996 to 2020 at Kyoto University Hospital, Japan, were analyzed using the Kaplan‐Meier method. The diagnosis of NASH was proven based on pathological findings of liver biopsy. After LT, all patients were examined at least every 3‐6 months with more frequent visits when clinically indicated. Blood tests and abdominal ultrasonography were performed on each of these visits, and computed tomography was scheduled when indicated. Liver biopsy was scheduled when abnormal liver function and/or hepatic steatosis were indicated. The cumulative incidence rate of (A) recurrent or de novo NAFLD and (B) recurrent or de novo NASH after LT for non‐B non‐C liver disease. *log‐rank test. HCC, hepatocellular carcinoma; LT, liver transplantation; NAFLD, nonalcoholic fatty liver disease; NASH, nonalcoholic steatohepatitis.

Here, we exemplify the occurrence of post‐LT NAFLD/NASH by analyzing 101 recipients who underwent LT for non‐B non‐C cirrhosis and HCC (excluding AIH, PBC, PSC, and cases with overlapping etiologies) from 1996 to 2020 at Kyoto University Hospital, Japan. For definitive diagnosis of NASH recurrence, liver biopsy was performed in patients with abnormal liver function after LT or those with fatty liver indicated by imaging studies, including ultrasonography and/or computed tomography. The diagnosis of NAFLD/NASH was proven based on pathological findings.^81^, ^82^ The cumulative 1‐, 3‐, 5‐, and 10‐year incidence rates for post‐LT recurrent NAFLD and recurrent NASH were 27%, 63%, 63%, and 88% and 23%, 52%, 52%, and 58%, respectively, in NASH recipients (*N* = 26). Those for de novo NAFLD and de novo NASH were 23%, 31%, 37%, and 40% and 13%, 16%, 18%, and 22%, respectively, in non‐NASH recipients, including ALD (*N* = 52) and cryptogenic (*N* = 23) cases (Figure 3).

## 6 SURVEILLANCE AND TREATMENT AFTER LIVER TRANSPLANTATION FOR HCC

HCC recurrence after liver transplantation peaks within 2 to 3 years after LT. The most common recurrence pattern is extrahepatic metastasis in lung and bone. Early recurrence within 1 year after LT could occur due to undetected extrahepatic metastasis that might present prior to transplantation or circulating cancer cells engrafting and growing in the target sites.^11^ Although there are no evidence‐based recommendations for posttransplant surveillance specifically after LT for HCC, imaging examinations using computed tomography and magnetic resonance imaging and measurements of tumor markers every 6 months are performed in a manner based on general HCC surveillance.^26^

Immunosuppressive regimens after LT are associated with HCC recurrence. CNIs are one of the standard agents used after LT; however, from an oncological point of view, CNIs reportedly promote tumor growth.^83^ Alternatively, mTORi inhibit the growth of cancer cells by modifying mTOR signaling, which plays a fundamental role in cell metabolism and proliferation.^84^ Although reducing the dose of CNI and adding a mTORi may reduce the risk of HCC recurrence,^85^ a randomized controlled trial (SiLVER study)^86^ resulted in limited advantages of mTORi in the prevention of post‐LT tumor recurrence, and further validation may be needed. The benefits of CNI reduction and the use of mTORi have been noted from the perspective of renal protection, while the risk of adverse effects of mTORi, such as hepatic artery thrombosis and delayed wound healing, should be taken into consideration.^87^ The use of corticosteroids is also associated with HCC recurrence due to cancer immune suppression. Using a lowered dose of steroids is preferable from the viewpoint of not only preventing tumor recurrence but also reducing metabolic dysfunction‐related risks, particularly in LT for NAFLD/NASH‐related HCC.^66^ Despite the requirements to establish a regimen that balances the risk of rejection and cancer recurrence, there are no randomized controlled trials or other studies with a high level of evidence at present. In non‐B non‐C HCC, the comorbidities related to underlying diseases and the biological behavior of HCC are diverse, and thus the development of individualized immunosuppressant protocols and the investigation of biomarkers of immune tolerance for weaning of immunosuppressants remain challenging.

There is no sufficient evidence of the efficacy of adjuvant therapy after LT for HCC. For example, adjuvant therapy using sorafenib did not contribute to recurrence‐free or overall survival, showing that its benefits were limited.^88^ Although molecular‐targeted drugs have made significant advances in therapeutic strategies for HCC in recent years, their efficacy in preventing recurrence after LT has yet to be verified.

The effectiveness of surgical resection, locoregional therapy including transcatheter arterial chemoembolization and radiofrequency ablation, and systemic therapy such as sorafenib has been reported as treatment after recurrence.^11^, ^88^ R0 resection in both intrahepatic and extrahepatic recurrences is expected to improve prognosis.^89^ However, these data were limited, and long‐term improvement in prognosis could only be achieved in rare cases.

Immune checkpoint inhibitors (ICIs) have made remarkable progress in recent years as a treatment option for advanced HCC; on the other hand, ICIs increase the risk of rejection and graft loss after LT, and there is no evidence that the antitumor effect outweighs the risk of rejection.^11^, ^88^ The washout period between the last dose of ICI and LT is an essential factor to consider to avoid the potential risk of acute rejection after LT.^90^, ^91^ One of the current large series of patients with ICI use prior to LT suggested that pretransplant use of ICIs within 90 days of LT was associated with biopsy‐proven acute cellular rejection and immune‐mediated hepatic necrosis.^92^ Several case reports support the validity of a withdrawal period of 90 days or more,^93^, ^94^ and a case with a short washout period of 8 days resulted in fatal acute hepatic necrosis.^95^ On the other hand, a case series reported no increase in rejection or graft injury, although 89% of patients received ICI treatment within 4 weeks of LT.^96^ In contrast, one case with fatal acute hepatic necrosis was observed, and this outcome is likely related to the acute immune rejection caused by the pre‐LT ICI treatment despite 93 days of withdrawal.^97^ Overall, it is possible that a short interval between ICI infusion and transplant increases the risk of acute rejection postoperatively, and a 90‐day wash‐out period potentially represents a factor that can ensure safety. However, the quality of evidence remains limited in the research phase, these findings need further verification in multicentered prospective studies. It would also be important to explore biomarkers that could predict the risk of graft rejection and loss, such as PD‐1 and PD‐L1 expression.^95^, ^98^ From the oncological viewpoint of ICI use, it was suggested that atezolizumab + bevacizumab yielded no overall survival (OS) benefit compared to sorafenib for patients with nonviral HCC (median OS 17.0 vs 18.1 months) according to subgroup analysis of the IMbrave150 trial.^99^, ^100^ This differential activity appeared to be mainly driven by the unexpectedly good prognosis of the nonviral group in the sorafenib arm (median OS 18.1 months in the nonviral group, 13.4 months in the overall sorafenib arm) compared to the atezolizumab + bevacizumab arm (median OS 17 months in the nonviral group, 19.2 months in the overall atezolizumab + bevacizumab arm) in the IMbrave150 study. Thus, it has been suggested that the good performance of sorafenib in the nonviral group may have offset the difference in the risk, and the effect of ICI regimens in the nonviral group warrants further investigation.^101^, ^102^ A recent meta‐analysis of 3 phase III trials comprising a total of 1656 patients with unresectable HCC found that immunotherapy improved OS in patients with viral HCC but not in patients with nonviral HCC.^103^ The major limitation is that this meta‐analysis included both ICI monotherapy and combination treatment. Furthermore, nonviral etiologies remain heterogeneous, and subdivision into ALD and NAFLD/NASH is needed. Given the currently available data, it is impossible to draw conclusions regarding the efficacy of the currently approved ICI regimens for different HCC etiologies. Overall, despite the existing data on responses to ICI therapy for pre‐LT patients in bridging therapy settings, further investigations are required to demonstrate the oncologic benefits of the use of ICIs in pre‐ and post‐LT settings,^90^, ^92^, ^104^ especially in LT recipients with non‐B non‐C etiologies.

## 7 SUMMARY

Recently, the cases of LT for non‐B non‐C cirrhosis and HCC have been increasing in Japan as well as worldwide. Perioperative management of non‐B non‐C liver disease is problematic due to various risk factors, including alcohol consumption and metabolic syndrome‐related risks, such as obesity and diabetes mellitus. Stratifying the indications for LT in individual etiology based on the risk assessment for recurrence of the primary disease remains a future challenge.

## DISCLOSURE

Conflict of Interest: The authors declare no conflicts of interest for this article.

Ethical Approval: The analysis of human data in this study was performed in accordance with the ethical guidelines for epidemiological research in Japan and was approved by the Ethics Committee of the Kyoto University Graduate School and Faculty of Medicine (approval code: E1473‐3). Written informed consent was obtained from all study patients.
